# Ban-It-Harderism in European Consumer Law: The Case of the French Influencer Law

**DOI:** 10.1007/s10603-025-09600-6

**Published:** 2025-09-23

**Authors:** Laura Aade, Catalina Goanta

**Affiliations:** 1 University of Luxembourg, Luxembourg, Luxembourg; 2 Utrecht University, Utrecht, Netherlands

**Keywords:** European consumer protection, Influencer marketing, Unfair Commercial Practices Directive, Hidden advertising

## Abstract

In June 2023, France adopted a new law to curb the negative impact of influencer marketing on its consumers. While it introduces some innovative provisions, the law mostly doubles down on legal prohibitions that could have been equally construed under existing consumer protection legislation. From this perspective, it can be argued that this law is an example of *ban-it-harderism*: a regulatory trend where lawmakers respond to perceived enforcement failures, not by addressing structural or institutional issues, but by enacting additional prohibitions that duplicate existing norms. Ban-it-harderism is problematic for at least two reasons. First, it often gives the false public impression that certain issues were not covered by regulation before the specific intervention. Second, it contributes to the overwhelming regulatory inflation seen at European and national levels on matters dealing with technology regulation and the digital market. This article discusses selected provisions from the French Influencer Law to contrast them with existing European consumer law and to critically reflect on its contributions to relevant legal frameworks.

## Introduction

In June 2023, France adopted a new law to curb the negative impact of influencer marketing on its consumers (Loi n° 2023-451, 2023; hereafter the French Influencer Law). This legislative development did not arise in a vacuum: it followed a series of high-profile public scandals, repeated concerns about hidden advertising, and a growing perception that influencer marketing operates in a regulatory grey zone (Le Point, 2022). Prior to the adoption of the law, the French consumer protection authority (Direction générale de la concurrence, de la consommation et de la répression des fraudes—hereafter DGCCRF) had already investigated over 60 influencers, revealing that 60% had failed to comply with advertising disclosure rules (Ministère de l’économie, des finances et de la souveraineté industrielle et numérique, 2023a). Simultaneously, a large-scale public consultation gathered input from nearly 19,000 participants on a vast array of issues, ranging from misleading commercial practices to the role of influencer agents (Ministère de l’économie, des finances et de la souveraineté industrielle et numérique, 2023b). In March 2024, just under a year after the adoption of the French Influencer Law, a report evaluating its implementation was released and presented before the National Assembly, highlighting both its strengths and areas for improvement in the future (Assemblée nationale, 2024).

With France promoting its law as an innovation to be followed by other jurisdictions as well, this article offers a more critical take. Our core argument is that for its consumer part, the French Influencer Law largely reiterates pre-existing obligations under EU consumer law, particularly those enshrined in the Unfair Commercial Practices Directive (hereafter UCPD), rather than introducing genuinely new regulatory safeguards. From this perspective, it can be argued that this law is an example of *ban-it-harderism*: a regulatory trend where lawmakers respond to perceived enforcement failures, not by addressing structural or institutional issues, but by enacting additional prohibitions that duplicate existing norms (The Economist, 2024).

Ban-it-harderism is problematic for at least two reasons. First, it risks creating the false public impression that certain issues were not covered by regulation before the specific intervention. In influencer marketing, a lot of the industry still echoes the incorrect assumption that simply because a law does not explicitly refer to influencers, there is no law covering influencers (Hund, 2024). This can lead and has led to a wrong market perception that influencer marketing is a Wild West, where technological innovation outpaced the development and application of the law. In a perceived legal vacuum, relevant actors believe that they are bound to fewer obligations than those actually imposed by the rich body of European consumer law developed over the past 50 years. Second, it contributes to the crowded space of technology and digital market regulation, increasing legal complexity without necessarily improving enforcement outcomes.

For European consumer policy, ban-it-harderism reveals a new layer of complexity. In recent times, a lot of concerns have been raised about the identity and coherence of consumer public policy due to the increasing fragmentation caused by a growing digital *acquis* (de Elizalde, 2025; Namysłowska, 2025). In addition to this emerging problem faced by a sector already characterised by a piecemeal approach to public policy, ban-it-harderism as described in this paper shows that further coherence issues should also be expected to increase at national level, with Member States overcompensating for the lack of clarity of EU law.

Our article proceeds as follows. We first briefly characterise the status quo of influencer marketing as a means of content monetisation, describe the legal context in which it emerged, and provide a brief overview of the regulatory momentum around influencer marketing in the European Union. We then discuss selected provisions from the French Influencer Law to contrast them with existing European consumer law. Next, we elaborate on ban-it-harderism in the context of influencer marketing and discuss its characteristics, namely the ineffectiveness of regulation and the regulatory first-mover status. We finally conclude with reflections on whether such specific laws, such as the French Influencer Law, complement or overlap with relevant legal frameworks at the European level.

## Regulators Turning Their Attention to Influencers

Content monetisation is fundamentally changing social media. Initially considered a space for peer socialisation within individual networks, social media has become home to a blossoming economy around content creation, expected to reach half a trillion dollars by 2027 (Goldman Sachs, 2023). Content monetisation consists in the ability of social media users—often called influencers or content creators—to generate revenue by posting content and engaging in various business models around advertising, direct selling or providing digital content and services to their audiences.

The appeal of influencers is that they are authentic Internet figures of different types and sizes who establish parasocial relations with their audiences based on authenticity, relatability, trust and aspiration (Lou & Kim, 2019). Globally, the influencer marketing industry alone, focused on advertising, has been booming during the past decade and is expected to grow to $22.2b in 2025 (Statista, 2022). Due to complex factors such as perceived audience impact and algorithmic platform governance (Bishop, 2019, Bishop, 2021a), influencers develop personal narrative strategies around advertising, which shape their online discourse (Enke & Borchers, 2021).

One of the most visible issues around influencer marketing has initially emerged from the promotion of products and services without disclosing the material connection behind such ads (Ershov & Mitchell, 2020). Consumers subsequently trust the advice of their favourite influencers based on the emotional links unilaterally built with those persons, called parasocial relations (Tolbert & Drogos, 2019), which can easily be abused for commercial gain. Such an impact has been repeatedly labelled as a consumer harm by both the European Parliament and the European Consumer Association (hereafter BEUC) (Michaelsen et al., 2022). It is also noteworthy that hidden advertising is not a disruptive repercussion of the use of social media, but that earlier iterations of consumer manipulation in advertising bear the same characteristics: commercial parties try to deceive consumers in thinking that products belong to (multi)media content, when in fact advertisers try to subliminally influence consumer decision-making. Earlier examples include product placement and advertorials (Gibbons & Katsirea, 2012; Sanchez Ruiz, 2017).

The negative effect of hidden advertising had not gone unnoticed by the regulators of other decades. In the European Union, the Unfair Commercial Practices Directive (hereafter UCPD), already adopted in 2005, crystalised prohibitions against such subliminal influence, whether in explicit terms (e.g., Point 11 of the Annex)^1^ or in general terms (e.g., the misleading test in Arts. 6–7 or the general unfairness test in Art. 5). Although the Directive could not have possibly had the foresight as to make direct reference to influencers in 2005, there is general consensus that it is directly applicable to this economic sector, as the European Commission (2021) acknowledges in its UCPD Guidance, in spite of remaining concerns about the complexity of enforcement. Beyond explicitly addressing earlier forms of influencer marketing, such as advertorials and product placement, the UCPD’s open test structure should have been, in the end, its finest feature: future-proofing rules that do not need updating every few years, but that would withstand the test of time and fit emerging legal problems.

In spite of (or perhaps due to) its openness, national authorities enforcing the UCPD have failed to signal its fitness in governing influencer advertising, which led to a regulatory frenzy in different Member States, but also at European level. In the past years, influencer marketing has been an important policy topic for both the European Commission and national lawmakers. For example, Spain adopted a law specifically targeting content creators who meet certain economic thresholds or audience sizes in 2024. Unlike the French initiative, this law does not apply broadly to all influencers. Instead, it imposes obligations only on those who meet specific thresholds—such as earning over €300,000 annually or having more than 2 million followers across platforms—thereby subjecting them to European media law (Santos, 2024). As already mentioned, the European Commission (2021), for its part, addressed influencer marketing in the comprehensive guidelines on the interpretation and application of the UCPD, clarifying issues such as the legal qualification of influencers as traders. However, due to the non-binding nature of these guidelines and the absence case law from the Court of Justice of the European Union (hereafter CJEU), the European Commission (2024a) noted in its Digital Fairness Fitness Check report that considerable legal uncertainty remains about the applicable rules to influencer marketing—particularly regarding the responsibilities of other actors in the advertising supply chain, including brands and platforms. To further investigate industry practices, the European Commission (2024b) coordinated a sweep on influencer practices in 22 Member States, revealing that a mere 20% of the posts of 576 investigated influencers systematically disclosed commercial content. In response, it is expected to propose targeted amendments to the UCPD, including explicit prohibitions and harmonised definitions of sector-specific terminology, in order to streamline disclosure practices and reduce regulatory fragmentation across the EU.

## Introducing the New French Influencer Law

The French Influencer Law is structured along two titles, respectively divided into three and two chapters. Title I (Articles 1–9) covers the nature of commercial influence by electronic means and obligations relating to its exercise. In particular, Articles 1 and 2 reflect general provisions relating to the activity of commercial influence by electronic means, while Articles 3–6 outline specific provisions relating to the promotion of goods and services as part of the activity of commercial influence by electronic means. Articles 3 and 4 list prohibitions on the promotion of certain goods and services, and Articles 5 and 6 mandate information duties with respect to the promotion of certain goods and services. Influencer agents, electronic commercial contracts, and further liability and insurance matters are covered by Articles 7–9. Title II (Articles 10–18) deals with the regulation of content published by persons involved in electronic commercial influence (in other words, social media platform content moderation obligations), as well as education activities for the youth. Articles 10–15 initially dealt with the regulation of content disseminated by influencers digitally,^2^ while Articles 16–18 address measures to raise public awareness for electronic commercial content.

In general, this law does not bring any groundbreaking changes to the French legal landscape in terms of the main obligations associated with influencer harms, namely curtailing some commercial practices and focusing on disclosures. It rather generally comprises amendments to existing national regulation on public health law, consumer protection, contract law, and platform liability. However, a few provisions do reflect some degree of innovation, such as definitions, interpretations, and additional liabilities set out in influencer marketing.

The French Influencer Law is supported by a Code of Practice (hereafter CoP), intended for influencers and content creators covered by the new law (Ministère de l’économie, des finances et de la souveraineté industrielle et numérique, 2023b). The CoP serves as a practical guide, clearly outlining their rights and obligations, as well as the ethical standards associated with their activities, in a clear and accessible manner. It also provides guidance and clarifications on specific articles contained in the French Influencer Law. However, several provisions of the French Influencer Law remain incomplete, as their application is contingent upon adopting decrees to be issued by the Council of State. As of this writing, only one of the five expected decrees has been adopted (Décret n° 2023-887, 2023). Relying on secondary legislation is particularly striking, given the objective of the law to clarify existing legal obligations. Instead of offering immediate legal certainty, it introduces a new layer of ambiguity, delaying the enforceability of its most detailed rules. This challenge is not new in the French legislative landscape: similar delays were observed with the adoption of decrees for the law regulating the activity of child influencers on online platforms adopted in 2020 (Loi n° 2020−1266, 2020). As such, the ambition of the French Influencer Law to clarify and harmonise rules is undermined by the very mechanisms intended to operationalise it.

In the next sub-sections, we explore some of the essential provisions of the French Influencer Law and reflect on their contribution to existing regulation. As emphasised above, the law comprises elements of consumer law, public health, private international law, and contract law. To shape a cohesive discussion about the content of the obligations, this paper focuses on those provisions relevant to consumer advertising.

### Legally Defining Influencers

France has made history by becoming the first country in the European Union to explicitly define the term “influencer” in a legal statute. The definition can be found in Article 1 of the French Influencer Law as follows: “any natural or legal person who, for a fee, uses their reputation among their audience to communicate content to the public by electronic means for the purpose of promoting, directly or indirectly, goods, services or any cause whatsoever.”^3^ Legally defining influencers is of great importance as it acknowledges their growing impact and role in modern society. Earlier definitions have been provided by the European Commission (2021) in its UCPD Guidance, or in academic work such as the comprehensive report on influencer marketing and consumer protection commissioned by the European Parliament (Michaelsen et al., 2022). The strength of the French definition lies in its inclusivity, encompassing the diverse business models used by influencers to monetise their content. Furthermore, the incorporation of political advertising (i.e., “any cause whatsoever”) within the definition demonstrates an understanding of the ever-evolving nature of influencer marketing (De Gregorio & Goanta, 2022).

But is this really a novelty without which European consumer law (e.g., the UCPD) would not be applicable to influencers? Certainly not. The main drawback of the existing European regime is the openness of the term “trader” which influencers are supposed to fall under. In the *Kamenova* case (Case C-105/17, 2018), the CJEU provided a list of non-exhaustive and non-exclusive criteria for national courts to interpret the notion of trader, such as the number, amount, and frequency of transactions, which is currently interpreted on a case-by-case basis at national level. In its UCPD Guidance, the European Commission (2021) specified that influencers who *frequently* (emphasis added) engage in promotional activities towards consumers on their social media accounts could legally qualify as traders, irrespective of their audience size. Since there are no explicit ceilings, in principle, even a very low threshold of economic activity could, under the *Kamenova* case (Case C-105/17, 2018), be interpreted to make microinfluencers who may earn €10k per year qualify, provided that it is frequent. Established megainfluencers who own multiple businesses are undoubtedly considered to be “traders.”

In France, according to the CoP, an individual falls within this definition as soon as financial or in-kind compensation is received to share commercial content online (Ministère de l’économie, des finances et de la souveraineté industrielle et numérique, 2023b). It is so far unclear how this standard will be applied in practice, given the broad array of business models and the nature of the industry, where most creators are not able to make a living out of influencer marketing and their revenue from it (including payment in natura) will often be highly volatile, inconsistent, and very limited. Will accepting leggings or protein bars as part of a barter agreement be enough for the French Influencer Law to trigger obligations? Most likely not, which shows a missed opportunity to clarify the “trader” concept with more concrete conditions.

### Prohibition to Promote Certain Goods and Services

A number of explicit prohibitions related to influencer marketing can be found in the French Influencer Law. For instance, Article 4-I strictly prohibits influencers from directly or indirectly promoting aesthetic procedures, processes, techniques, methods as well as surgeries. This provision is based on existing articles found in the French Public Health Code and aims to clarify their application to influencer marketing practices. As mentioned in Article 4-IX, a violation of this article carries a sanction of up to two years of imprisonment and a fine of up to €300k, to showcase the policy importance of health-related practices. This provision responds to multiple public scandals linked to the promotion of plastic surgeries and the subsequent increase in such procedures (Assemblée nationale, 2023). Some of these surgeries have had dramatic consequences, not only on vulnerable consumers but also on influencers themselves (Vidalie, 2022).

Another example of prohibitions found in the French Influencer Law concerns the promotion of gambling. According to Article 4-VII, any commercial content related to gambling, as defined in Articles L. 320-1 and L. 320-6 of the French Security Code, is prohibited unless the platform offers the possibility to restrict such content to children under 18 and this functionality is activated by influencers. In this case, influencers must still include a statement indicating that this content is prohibited for anyone under the age of 18, in addition to all the information listed in Title 1 of the French Internal Security Code. This notice must be clear, legible, as well as identifiable, in all formats, during the entire promotion, and aims to protect minors in instances where they falsify their age or use someone else’s account. Failure to comply with this article can result in a fine of €100k, as specified in Article L. 324-8-I of the French Internal Security Code.

### Obligation to Disclose Commercial and Synthetic Content

The French Influencer Law also addresses the obligation to disclose commercial content. Article 5-2 states that the promotion of goods, services, or any cause must be clearly disclosed with the labels “publicité” or “collaboration commerciale.” As mentioned in the CoP, influencers must also clearly tag the brand or person on whose behalf the commercial communication is being carried out (Ministère de l’économie, des finances et de la souveraineté industrielle et numérique, 2023b). Failure to comply with this obligation constitutes a misleading commercial practice by omission according to Article L. 121-3 of the French Consumer Code, transposing Article 7(2) UCPD. Influencers who engage in such unfair commercial practices are liable to 2 years of imprisonment and a fine of €300k, in accordance with Article L. 132-1 to L. 132-9 of the French Consumer Code. Should an influencer fail to comply with such sanctions, the DGCCRF can request social media platforms to implement measures aimed at curtailing the dissemination of content infringing consumer protection laws (Ministère de l’économie, des finances et de la souveraineté industrielle et numérique, 2023b). These measures may include displaying warning messages to consumers, shadowbanning (Leerssen, 2023), restricting or blocking access to a social media account. The DGCCRF also has the power to issue an order under penalty (*injunction sous astreinte*), imposing daily fines until the influencer complies with the sanction issued pursuant to Article L.521 of the French Consumer Code, as amended by Article 13 of the French Influencer Law.

At the request of the French government, social media platforms such as Snap, Instagram, and TikTok have adapted their in-app disclosure tools to align with the labels mentioned in the French Influencer Law (Annabell et al., 2024). When using these apps in French, influencers can label commercial content with the term “collaboration commerciale.” This transitioning from the toggle “paid partnership” to “collaboration commerciale” proves to be less misleading for influencers and their audiences, as the term “paid” could be construed to mean only sponsorships paid with money, when in fact there is a large exchange of goods and services in this industry. As confirmed by the European Commission (2021) in the UCPD Guidance and in light of the *Peek & Cloppenburg* case (Case C-371/20, 2021), any form of consideration received by the influencer for commercial content is considered payment, also in the case of affiliate marketing or barters (Luzak & Goanta, 2022).

Notably, the requirement that disclosures be made with a specific terminology in French marks a departure from existing EU regulations, which do not prescribe a language for such labels. While soft law instruments in some Member States suggest using the language of the content or target audience, France is the first country in the EU to codify this obligation in binding legislation. This raises important questions about legal fragmentation in the absence of EU-level harmonisation. Despite the existence of an EU-wide transparency obligation under the UCPD, the Digital Fairness Fitness Check highlighted considerable legal uncertainty about the required standards and modalities of advertising disclosures (European Commission, 2024a). Courts, regulators, and national guidelines diverge on critical aspects such as the exact terminology to be used, the language of disclosure, and the level of visual prominence required. The French approach reflects an attempt to bring clarity, but also illustrates the broader patchwork of disclosure obligations emerging across the EU.

In addition to disclosing commercial content, Article 5 of the French Influencer Law introduces another disclosure duty related to synthetic content. It essentially requires that commercial content including images which have been altered by any image-processing technique, aimed at slimming, thickening the silhouette, or modifying the appearance of the face, must be labelled with “images retouchées.” Additionally, commercial content including images produced by artificial intelligence to represent a face or silhouette must be assigned with the label “images virtuelles.” This provision is inspired by the existing *Loi Mannequin* (Décret n° 2017−738, 2017), requiring every picture of models used in advertising campaigns to be labelled with the words “photo retouchée” when the morphology of the body has been modified. Failure to disclose synthetic content in commercial communications is punishable by one year of imprisonment and a fine of €4,5k. All these labels must be clear, legible, and identifiable on the commercial content, in all formats, throughout the entire promotion. According to Article 5-IV of the French Influencer Law, the application requirements of these disclosure duties should have been specified in a decree by the end of 2023.

Although no comparable rule currently exists under the EU consumer *acquis*, as with the promotion of cosmetic surgery or gambling, editing photos without disclosure can also be considered an unfair commercial practice and would thus already be covered by the UCPD, to the extent that it relies on manipulating consumers to engage in the purchasing of goods or services. If an influencer promotes a beauty product or a weight loss product and then uses filters for smooth skin or body skinning, it shapes a dishonest reality. This may be a common practice in the professional advertising world, where advertisers enjoy some degree of discretion for mere puffs, but the social media world operates under different rules. The parasociality and relatability inherent to social media, paired with the perceived authenticity of influencer content, would make such a practice unfair, due to the fact that filters create idealised influencers whose real personas are very different (Forbrukerstilsynet, 2022).

### Extraterritorial Effect

The French Influencer Law is explicitly designed to apply extra-territorially and aims to cover influencers located outside the European Union, Switzerland or the EEA, if their content targets a French audience. As confirmed in the CoP, influencers based outside France can have their content blocked if they fail to comply with French law by, for instance, not disclosing commercial content or by advertising products whose promotion is regulated or forbidden (Ministère de l’économie, des finances et de la souveraineté industrielle et numérique, 2023b). This extra-territorial effect was prompted by the amount of influencers, particularly from reality TV shows, relocating from France to Dubai for various reasons (Ragot, 2023). To prevent them from bypassing their legal responsibilities, the French Influencer Law introduces two new obligations for these influencers, as outlined in Article 9.

First, Article 9-I of the French Influencer Law requires influencers established outside the EU to designate a natural or legal person to represent them within the EU. This legal representative must ensure that influencer agreements are properly performed, particularly those targeting an audience located in France. Additionally, that person must respond to requests from competent authorities or judicial bodies regarding the enforcement of the French Influencer Law. In turn, influencers must grant their legal representatives the necessary powers and resources to effectively cooperate with the competent authorities. They should also provide administrative authorities with the contact details of their legal representatives upon request. Second, influencers who are not established within the EU must subscribe to a civil liability insurance, as required by Article 9-II. The insurance provider must be based in the EU and is intended to cover potential damages caused to third parties as a result of influencer marketing services, without the need for a court decision. Article 9-III further states that the application of these two obligations to influencers based outside the EU will be defined by a decree. The latter was expected to be adopted by the end of 2023 or, at the latest, early 2024—which is still not the case at the time of writing.

Existing tests in consumer protection law focus on criteria used to determine if a trader directs “its activity to the Member State of the consumer’s domicile” (Chen, 2022). This determination can be made using evidence from the trader’s website, such as “mention of telephone numbers with the international code, use of a top-level domain name other than that of the Member State in which the trader is established, for example ‘.de’, or use of neutral top-level domain names such as ‘.com’ or ‘.eu’; […] and mention of an international clientele composed of customers domiciled in various Member States, in particular by presentation of accounts written by such customers,” as specified by the CJEU in the *Pammer* case (Case C-585/08, 2010). Although these tests have not yet been applied to social media advertising, there are no reasons to believe that they could not be moulded to new technical realities. A true innovation on this matter would have been a more explicit recognition of the role that online platforms play in determining who sees what content. On social media, influencers are not in a technology architecture position to determine who sees their content—that is the role of the platform. And this role remains opaque even in the French Influencer Law, which unfortunately does not contribute to the effective clarification of such implications.

## Ban-It-Harderism in Influencer Marketing

The selected provisions from the French Influencer Law discussed in the section illustrate that, with respect to consumer advertising, they do not introduce fundamentally new elements beyond what is already established under some provisions of the UCPD. What results is yet another law, passed with great haste and public acclaim, building on both national and European frameworks. Overall, the question then becomes: is this law a net benefit for regulatory interventions in this field—namely will it actually solve the problems posed by the influencer industry—or is it just another law that simply bans certain already banned aspects of the digital market harder?

In what follows, we critically reflect on the contribution brought by the French Influencer Law to the existing regulatory framework, by focusing on two particular points: the effectiveness of regulation and the regulatory first-mover status in the field of technology. These two aspects characterise ban-it-harderism as a regulatory phenomenon. Adopting apparently stricter rules instead of identifying the root causes of existing problems undermines the effectiveness of regulation, as the lawmaker pursues a policy goal of loudly reasserting existing laws rather than painstakingly troubleshooting institutional pitfalls—in this case particularly relating to enforcement. In turn, the regulatory first-mover status can improve public trust in governance, as the lawmaker is perceived to save consumers from looming online harms and subsequently also creates some degree of national pride in the exporting of seeming regulatory innovation.

### The Effectiveness of Regulation

The French Influencer Law is generally echoed by existing regulation. In this paper, we focused mainly on the overlap with the UCPD, but additional concerns exist with respect to the Audiovisual Media Services Directive and other European legislation relating to consumer health, as well as national contract law, or administrative law regimes. As we emphasised at the onset of this paper, influencer marketing is not a new phenomenon, but rather another iteration of the old problem of dishonesty towards consumers. Legal instruments such as the UCPD should be more than fit to govern old problems with new wrappings. The UCPD is a maximum harmonisation directive, meaning that Member States may not impose additional, more protective standards (Collins, 2004; Duivenvoorde, 2015; Durovic, 2016). Yet even this policy approach, focused on legal convergence, has achieved little in practice in the field of influencer marketing. The development of existing regulation into a compatible solution for influencer marketing has suffered on two fronts.

Firstly, there is a widespread interpretation issue that industries such as influencer marketing will always face. This advertising business model is part of a content monetisation shift that is often very difficult to follow, map, and describe. Social media platforms develop new functionalities with the speed of light, changing this market constantly and at an unprecedented pace. At the same time, marketing trends shift with the same speed—in a span of years, mega-influencers have been replaced in marketing campaigns by micro- and nano-influencers—and these are just some economic implications (Goanta & Ranchordás, 2020). In addition, influencer marketing is also a popular culture phenomenon that captures media trends reflecting deep transitions in our societies (Bishop, 2021b). This makes influencer marketing—and content creation in general—very complex to understand, particularly without experiencing it. In consequence, it is cumbersome for policymakers, judges, and regulatory agencies to be up to date with the monetisation status quo and thus, it becomes difficult to contextualise legal issues in this landscape. For this reason, additional regulation does not fix this issue of interpretation. Although the European Commission is currently working on the Digital Fairness Act and could potentially introduce specific rules for influencers, regulators must take a step back and ask themselves whether overly specific rules would not be immediately outdated. Instead of struggling to get UCPD or national law amendments every 2 years and still lagging behind the reality of business practices, relevant institutions could rely on up-to-date market research to get a grasp of the creator economy at a given moment in time. This can be achieved through specific guidelines, such as those issued for the UCPD, which can inspire further guidance at national level to tackle local preferences and issues. Alternatively, one inspiring aspect of the French reform is the introduction of codes of practice accompanying statutory provisions, towards the same goal: complementing open-ended, future-proof legislation with more specific and informed interpretations of market practices.

Secondly, the interpretation issue trickles down into a greater problem of enforcement. The UCPD is only an example of how statutory frameworks can be meaningless unless they are effectively applied in practice. Existing consumer protection legislation in France, rooted in the European *acquis*, has failed to govern influencer marketing not because it was not fit to do so, but rather because its enforcement structures were not equipped to deal with it. The activity of the French consumer enforcement agency (DGCCRF) proves this point. After several influencer scandals that weakened consumer trust, the DGCCRF started taking stronger enforcement actions against influencers (Ministère de l’économie, des finances et de la souveraineté industrielle et numérique, 2023c). Before the adoption of the French Influencer Law, the authority closely monitored the activities of several influencers and imposed sanctions on a number of them. It publicly disclosed the names of some content creators found to be involved in misleading commercial practices under the existing French Consumer Code. Some of these influencers were subsequently required to post their respective injunctions on their social media accounts for periods ranging from 15 to 30 days (Ministère de l’économie, des finances et de la souveraineté industrielle et numérique, 2023d). In the assessment report, the DGCCRF expressed its intention to significantly enhance its enforcements efforts, with more regular monitoring on influencers in 2024 (Assemblée nationale, 2024). As of this writing, it has already issued 27 warnings, 57 administrative injunctions, 17 criminal records, and imposed 3 administrative sanctions (Ministère de l’économie, des finances et de la souveraineté industrielle et numérique, 2023d). This proves that what stood in the way of applying existing consumer protection rules was not a lack of relevant regulation, but rather a lack of resources and prioritisation of influencer monitoring by relevant French authorities (Goanta, 2023). These enforcement actions were used by the French authority to justify its subsequent regulatory intervention. However, this justification is misplaced—what is instead necessary before a comprehensive reform is an assessment of the enforcement capacity, particularly when dealing with digital monitoring at scale.

### The Regulatory First-Mover Status

It seems commonplace nowadays that technology regulation emerges as a result of a public outcry against some perceived harms. With presentations in Madrid, London, and Brussels, the proponents of the French Influencer Law have presented this reform as a legal innovation that ought to inspire other jurisdictions as well. After all, the first-mover advantage is that whoever is first to enter the regulatory marketplace “has the upper hand to define what follows” (Wheeler, 2023). Implicitly, if other countries adopt the rules of a first mover, it can give the latter a step ahead, particularly in having already internalised transaction costs of new regulation. The French Influencer Law has set a resoundingly harsh tone emphasising the negative impact of influencers on consumers. While it is true that some of the far-reaching provisions of the law (e.g., criminal law imprisonment sanctions) have the reasonable purpose of deterring influencers from inducing grave harms on French consumers, its tone reflects influencer bashing tendencies. Influencer bashing entails the ridiculing of influencers, seen in a negative light by some parts of society (Pabian, 2022), instead of merely trying to limit the negative consequences of an otherwise innovative and economically growing activity. Influencer marketing and content creation have generated digital occupations with contested social value and reputation (Newlands & Lutz, 2024), particularly since mainstream media has been very generous in taxing influencer gaffes and mistakes – some of which rightfully problematic.

In France, regulatory reform was hastened—among others—due to the actions of public figures, such as the French rapper Booba who initiated a social media campaign to expose the scams and scandals prevalent within the influencer industry. His efforts were directed towards shedding light on the dubious practices employed by numerous influencers, particularly those emerging from reality TV shows. However, the main target of his campaign was Magali Berdah, a prominent businesswoman running the largest influencer marketing agency in France. To highlight the scale of the issue, Booba launched the hashtag #influvoleur and created a dedicated mailbox to gather testimonials from individuals claiming to have been victims of scams promoted by influencers. The constitutive elements of this public scandal can be traced in the French Influencer Law (e.g., the specific cosmetic surgery and gambling prohibitions, as well as the focus on influencer agents).

In the light of public scandals such as the ones just described, the French Influencer Law emerged as a promise to save the French public against the perceived atrocities and illegalities committed by predatory influencers. It can be argued that this approach has had both positive and negative effects. The positive effects consist of the sheer attention the reform has received, at a time when the DGCCRF was also becoming more proactive in addressing wrongdoing by influencers. The resulting naming and shaming of influencer practices (or actual influencers themselves) can have a short-term deterring effect for consumer harms inflicted by influencers. Then again, this was and could have been done by further interpreting existing regulation in a manner tailored to influencer marketing realities. By not doing so, the resulting negative effects include contributing to regulatory inflation and further complicating the already complex web of applicable laws. At the same time, influencer marketing and content creation in general can be digital occupations that, if nurtured well, could blossom into healthy economic ecosystems. A general negative tone against the wrongdoings of this industry that avoids the root problems can have a detrimental impact on its inherent innovation. In doing so, the French Influencer Law shows how lawmakers tend to wait for too long with policing certain industries and when the enforcement gap becomes too large, they retaliate with regulatory bans.

The French reform can now be taken as inspiration by other jurisdictions, to the satisfaction of the French lawmaker. However, perhaps the most problematic aspect of becoming a regulatory first-mover in this field is that national regulation will only make matters more complex and not really solve them. Influencer marketing remains a transnational phenomenon that needs supranational statutes and standards. The content of (and harms done by) French influencers go beyond physical borders and legal jurisdiction of France and yet the law remains very nationally oriented, which might be beneficial for French consumers, but not so much for the rest of the EU. There is no need for 27 influencer definitions and 27 enforcement regimes, but rather for streamlined guidelines for the interpretation of existing and harmonising European consumer legislation. It remains to be seen whether the EU’s ongoing reform of the UCPD will be inspired by the French Influencer Law.

## Conclusion: The Added Value of the French Influencer Law

All in all, the French Influencer Law introduces new rules but mainly builds on existing regulation. Its most essential substantive contributions are reiterations of existing prohibitions or rules on disclosures which can already be found in European consumer protection legislation. Essentially, the French Influencer Law simply bans some practices harder than they were banned before. However, the perceived success story of the French reform overshadowed its negative regulatory impact. Taking a harsh stance against influencers as a whole in order to identify and sanction the most predatory among them has left no room to discuss the benefits of influencer marketing—information literacy education (e.g., news commentary), the reduction of information asymmetry (e.g., honest product reviews), or creativity enhancement just to name a few. This complex market operates in a space that both European and national regulators failed to follow and comprehend, namely the monetisation of social media gig work. Here, one of the root causes of the problems posed by influencers is that their labour has not been accurately defined, classified, and supervised in the past decade and beyond. In spite of the rich debates around independent contracting and freelancing as a result of the shortcomings of the sharing economy, there is no harmonised European freelancer status in terms of legal personhood, no harmonised influencer definition, and no harmonised approach to platform liability. Indeed, the hyper-complex content creation economy and its embedding in the tricky and opaque social media platform governance create a heavy burden for lawmakers to figure out how to best disentangle complexities. Yet national solutions—as pungent as they may be—are not fit for transborder phenomena that go well beyond their jurisdiction, particularly when existing European frameworks have been around for decades to fix the same problems.

## Data Availability

No datasets were generated or analysed during the current study.

## Footnotes

1 Point 11 reads: “using editorial content in the media to promote a product where a trader has paid for the promotion without making that clear in the content or by images or sounds clearly identifiable by the consumer (advertorial)”.

2 Those articles were repealed by Law n° 2024-364 of 22 April 2024 after discussions with the European Commission, which flagged concerns over their incompatibility with European law. A leaked letter from former Commissioner Thierry Breton highlighted that these provisions overlapped with several DSA obligations, such as notice and action mechanisms, trusted flaggers, transparency reporting obligations, and orders to act against illegal content, as respectively found in Articles 9, 10, 15, 16, and 22 DSA. The Commission warned that this could breach the country-of-origin principle, as French authorities claimed jurisdictions over intermediary services providers outside France, thus violating Article 56 DSA.

3 The original version of the definition reads: “Les personne physiques ou morales qui, à titre onéreux, mobilisent leur notoriété auprès de leur audience pour communiquer au public par voie électronique des contenus visant à faire la promotion, directement ou indirectement, de biens, de services ou d’une cause quelconque exercent l’activité d’influence commerciale par voie électronique.”

## References

[CR1] Annabell, T., Aade, L., & Goanta, C. (2024). (Un)disclosed brand partnerships: How platform policies and interfaces shape commercial content for influencers. *Internet Policy Review, 13*(4). 10.14763/2024.4.1814

[CR2] Assemblée nationale. (2023). *Rapport fait au nom de la commission des affaires économiques sur la proposition de loi visant à lutter contre les arnaques et les dérives des influenceurs sur les réseaux sociaux.* Retrieved April 12, 2024, from https://www.assemblee-nationale.fr/dyn/16/rapports/cion-eco/l16b1006_rapport-fond

[CR3] Assemblée nationale. (2024). *Rapport d'information déposé en l'application de l'article 145–7 du Règlement par la Commission des affaires économiques sur l'application de la loi* n° 2023–451 du 9 juin 2023 visant à encadrer l’influence commerciale et à lutter contre les dérives des influenceurs sur les réseaux sociaux. Retrieved April 23, 2024, from https://www.assemblee-nationale.fr/dyn/16/rapports/cion-eco/l16b2339_rapport-information

[CR4] Bishop, S. (2021b, June 14). *Name of the game: “Creator” and “influencer” aren’t different jobs. Who does it serve to pretend they are?* Real Life. Retrieved September 18, 2023, from https://reallifemag.com/name-of-the-game/

[CR5] Bishop, S. (2019). Managing visibility on YouTube through algorithmic gossip. *New Media & Society,**21*(11–12), 2589–2606. 10.1177/1461444819854731

[CR6] Bishop, S. (2021a). Influencer management tools: Algorithmic cultures, brand safety, and bias. *Social Media + Society*,* 7*(1). 10.1177/20563051211003066

[CR7] Chen, Z. (2022). *Electronic consumer contracts and private international law: Combining targeting test with dis-targeting test.* European Association of Private International Law. Retrieved August 2, 2024, from https://eapil.org/2022/01/24/electronic-consumer-contracts-and-private-international-law-combining-targeting-test-with-dis-targeting-test/

[CR8] Collins, H. (2004). *The forthcoming EC directive on unfair commercial practices*. Kluwer Law International.

[CR9] European Commission. (2021). *Commission notice - Guidance on the interpretation and application of Directive 2005/29/EC of the European Parliament and of the Council concerning unfair business-to-consumer commercial practices in the internal market.* OJ C 526/1.

[CR10] European Commission. (2024b). *Investigation of the Commission and consumer authorities finds that online influencers rarely disclose commercial content.* Retrieved March 5, 2024, from https://ec.europa.eu/commission/presscorner/detail/en/ip_24_708

[CR11] European Commission. (2024a). *Commission staff working document fitness check on EU consumer law on digital fairness.* SWD(2024) 230 final. Retrieved July 29, 2024, from https://ec.europa.eu/info/law/better-regulation/have-your-say/initiatives/13413-Digital-fairness-fitness-check-on-EU-consumer-law

[CR12] de Elizalde, F. (2025). Fragmenting consumer law through data protection and digital market regulations: The DMA, the DSA, the GDPR, and EU consumer law. *Journal of Consumer Policy*. 10.1007/s10603-025-09584-3

[CR13] De Gregorio, G., & Goanta, C. (2022). The influencer republic: Monetizing political speech on social media. *German Law Journal,**23*(2), 204–225. 10.1017/glj.2022.15

[CR14] Duivenvoorde, B. B. (2015). *The consumer benchmarks in the unfair commercial practices directive*. Springer.

[CR15] Durovic, M. (2016). *European law on unfair commercial practices and contract law*. Hart Publishing.

[CR16] Enke, N., & Borchers, N. S. (2021). Social media influencers in strategic communication: A conceptual framework for strategic social media influencer communication. In N. S. Borchers (Ed.), *Social media influencers in strategic communication* (1^st^ edition). Routledge.

[CR17] Ershov, D., & Mitchell, M. (2020). The effects of influencer advertising disclosure regulations: Evidence from Instagram. *Proceedings of the 21st ACM Conference on Economics and Computation*, 73–74. 10.1145/3391403.3399477

[CR18] The Economist. (2024, February 14). *Ban it harder! An unwelcome new trend in British politics.* The Economist. Retrieved August 2, 2024, from https://www.economist.com/britain/2024/02/14/ban-it-harder-an-unwelcome-new-trend-in-british-politics

[CR19] Forbrukersilsynet. (2022). *The Norwegian Consumer Authority’s guideline on labelling retouched advertising.* Forbrukersilsynet*.* Retrieved May 21, 2025, from https://www.forbrukertilsynet.no/english/guidelines/the-norwegian-consumer-authoritys-guideline-on-labelling-retouched-advertising

[CR20] Gibbons, T., & Katsirea, I. (2012). Commercial influences on programme content: The German and UK approaches to transposing EU rules on product placement broadcasting in an age of commercialism. *Journal of Media Law,**4*(2), 159–188.

[CR21] Goanta, C. (2023). Digital detectives: A research agenda for consumer forensics. *European Papers - A Journal on Law and Integration*, *2023 8*(2), 647–663. 10.15166/2499-8249/680

[CR22] Goanta, C., & Ranchordás, S. (2020). The regulation of social media influencers. *Edward Elgar Publishing. Doi,**10*(4337/9781788978286), 00008.

[CR23] Hund, E. (2024, May 1). *Why the influencer industry needs guardrails and how to professionalize a maturing practice*. Harvard Business Review. Retrieved August, 2, 2024, from https://hbr.org/2024/05/why-the-influencer-industry-needs-guardrails

[CR24] Leerssen, P. (2023). An end to shadow banning? Transparency rights in the Digital Services Act between content moderation and curation. *Computer Law & Security Review,**48*, Article 105790. 10.1016/j.clsr.2023.105790

[CR25] Lou, C., & Kim, H. K. (2019). Fancying the new rich and famous? Explicating the roles of influencer content, credibility, and parental mediation in adolescents’ parasocial relationship, materialism, and purchase intentions. *Frontiers in Psychology,**10*, 1–17. 10.3389/fpsyg.2019.02567 31803110 10.3389/fpsyg.2019.02567 PMC6872518

[CR26] Luzak, J., & Goanta, C. (2022). Influencer disclosure obligations in the aftermath of Peek and Cloppenburg. *Journal of European Consumer and Market Law,**11*(5), 188–191.

[CR27] Michaelsen, F., Collini, L., Jacob, C., Goanta, C., Kettner, S., Bishop, S., Hausamer, P., Thorun, C., & Yesiloglu, S. (2022). *The impact of influencers on advertising and consumer protection in the Single Market*. European Parliament. Retrieved March 6, 2024, from https://www.europarl.europa.eu/thinktank/en/document/IPOL_STU(2022)703350

[CR28] Ministère de l’économie, des finances et de la souveraineté industrielle et numérique. (2023a). *Marketing d'influence: 60% des influenceurs ciblés par la DGCCRF en anomalie.* Retrieved February 27, 2024, from https://www.economie.gouv.fr/actualites/60-des-influenceurs-et-des-createurs-de-contenus-controles-par-la-dgccrf-concernes-par

[CR29] Ministère de l'économie, des finances et de la souveraineté industrielle et numérique. (2023b). *Guide de bonne conduite: influenceurs et créateurs de contenu.* Retrieved February 27, 2024, from https://www.economie.gouv.fr/guide-bonne-conduite-influenceurs-createurs-contenu

[CR30] Ministère de l'économie, des finances et de la souveraineté industrielle et numérique. (2023c). *Marketing d'influence: La DGCCRF intensifie ses contrôles au premier trimestre 2023.* Retrieved February 27, 2024, from https://www.economie.gouv.fr/dgccrf/marketing-dinfluence-la-dgccrf-intensifie-ses-controles-au-premier-trimestre-2023-0

[CR31] Ministère de l'économie, des finances et de la souveraineté industrielle et numérique. (2023d). *Publication des sanctions and injonctions: un outil très efficace pour informer les consommateurs et dissuader les professionnels indélicats.* Retrieved February 27, 2024, from https://www.economie.gouv.fr/dgccrf/publication-des-sanctions-et-injonctions-un-outil-tres-efficace-pour-informer-les

[CR32] Namysłowska, M. (2025). The silent death of EU consumer law and its resilient revival: Reinventing consumer protection against unfair digital commercial practices. *Journal of Consumer Policy*. 10.1007/s10603-025-09590-5

[CR33] Newlands, G., & Lutz, C. (2024). Mapping the prestige and social value of occupations in the digital economy. *Journal of Business Research,**180*, Article 114716. 10.1016/j.jbusres.2024.114716

[CR34] Pabian, S. (2022). *Defending my online friend: Investigating bystander reactions on influencer bashing among female Instagram users.* 72nd Annual International Communication Association (ICA) Conference. Retrieved March 5, 2024, from https://repository.uantwerpen.be/docman/irua/fded9a/189311.pdf

[CR35] Le Point. (2022, July 29). *Le rappeur Booba accuse des influenceurs d'escroqueries.* Le Point. Retrieved August 2, 2024, from https://www.lepoint.fr/societe/le-rappeur-booba-accuse-des-influenceurs-d-escroqueries-29-07-2022-2484839_23.php

[CR36] Ragot, J. (2023, August 17). *Influenceurs: Résider à Dubaï protège-t-il vraiment de la loi française?* BFMTV. Retrieved August 2, 2024, from https://www.bfmtv.com/tech/actualites/reseaux-sociaux/resider-a-dubai-protege-t-il-vraiment-les-influenceurs-de-la-loi-francaise_AV-202308170442.html

[CR37] Goldman Sachs. (2023, April 19). *The creator economy could approach half-a-trillion dollars by 2027.* Goldman Sachs. Retrieved March 5, 2024, from https://www.goldmansachs.com/insights/articles/the-creator-economy-could-approach-half-a-trillion-dollars-by-2027

[CR38] Sanchez Ruiz, M. (2017). The current European rules governing product placement and the new legislative proposal amending the Audiovisual Media Services Directive estudios. *Cuadernos de Derecho Transnacional,**9*(2), 506–529.

[CR39] Santos, A. (2024, April 30). *Government approves new social media ‘influencers law’ in Spain and details the content creators it will apply to*. Sur in English. Retrieved August 2, 2024, from https://www.surinenglish.com/spain/the-government-approves-the-law-influencers-control-20240430161931-nt.html

[CR40] Statista. (2022). *Global influencer market value 2020–2025.* Retrieved August, 2, 2024, from https://www.statista.com/statistics/1328195/global-influencer-market-value/

[CR41] Tolbert, A. N., & Drogos, K. L. (2019). Tweens’ wishful identification and parasocial relationships with YouTubers. *Frontiers in Psychology*. 10.3389/fpsyg.2019.02781 10.3389/fpsyg.2019.02781 PMC6928007 31920829

[CR42] Vidalie, A. (2022, August 17). *The ugly side of aesthetic medicine, an industry plagued by charlatans.* Le Monde. Retrieved August 2, 2024, from https://www.lemonde.fr/en/summer-reads/article/2022/08/17/the-ugly-side-of-aesthetic-medicine-an-industry-plagued-by-charlatans_5993904_183.html

[CR43] Wheeler, T. (2023, March 8). *The UK and EU establish positions as regulatory first movers while the US watches.* Brookings. Retrieved August 2, 2024, from https://www.brookings.edu/articles/the-uk-and-eu-establish-positions-as-regulatory-first-movers-while-the-us-watches/

[CR44] Directive 2005/29/EC of the European Parliament and of the Council of 11 May 2005 concerning unfair business-to-consumer commercial practices in the internal market and amending Council Directive 84/450/EEC, Directives 97/7/EC, 98/27/EC and 2002/65/EC of the European Parliament and of the Council and Regulation (EC) No 2006/2004 of the European Parliament and of the Council (‘Unfair Commercial Practices Directive’) (2005) OJ L 149/22

[CR45] *Komisia za zashtita na potrebelite v Evelina Kamenova* (Case C-105/17) ECLI:EU:C:2018:808

[CR46] Directive (EU) 2018/1808 of the European Parliament and of the Council of 14 November 2018 amending Directive 2010/13/EU on the coordination of certain provisions laid down by law, regulation or administrative action in Member States concerning the provision of audiovisual media services (Audiovisual Media Services Directive) in view of changing market realities (2018) OJ L 303

[CR47] *Peter Pammer v Reederei Karl Schlüter GmbH & Co. KG* (Case C-585/08) and *Hotel Alpenhof GesmbH v Oliver Heller* (Case C-144/09) ECLI:EU:C:2010:740

[CR48] *Peek & Cloppenburg KG, v Peek & Cloppenburg KG* (Case C-371/20) ECLI:EU:C:2021:520

[CR49] Décret n° 2017–738 du 4 mai 2017 relatif aux photographies à usage commercial de mannequins dont l'apparence corporelle a été modifiée

[CR50] Loi n° 2020–1266 du 19 octobre 2020 visant à encadrer l’exploitation commerciale de l’image d’enfants de moins de seize ans sur les plateformes en lignes

[CR51] Décret n° 2023–887 du 20 septembre 2023 relatif à la liquidation des astreintes prononcées en application des articles L. 521–1 et L. 521–2 du code de la consommation, et de l’article L. 470–1 du code de commerce

[CR52] Loi n° 2023–451 du 9 juin 2023 visant à encadrer l’influence commerciale et à lutter contre les dérives des influenceurs sur les réseaux sociaux

